# Weighted total genotype score associated with knee ligament injury incidence in adolescent female basketball athletes

**DOI:** 10.1186/s13102-026-01884-6

**Published:** 2026-07-14

**Authors:** Yumi Fukuyama, Yosuke Yamato, Yasushi Shinohara, Haruka Murakami, Motoyuki Iemitsu

**Affiliations:** 1 Faculty of Sport and Health Science, Ritsumeikan University, 1-1-1 Noji-higashi, Kusatsu, Shiga 525-8577 Japan; 2 Department of Physical Therapy, Aino University, 4-5-4 Higashioda, Ibaraki, Osaka 567-0012 Japan

**Keywords:** Knee ligament injury, Sex difference, Sports injury, Single nucleotide polymorphisms, Weighted total genotype score

## Abstract

**Background:**

Among the genetic factors of knee ligament (KL) injury, single-nucleotide polymorphisms (SNPs) in genes encoding proteins involved in ligament structure and function have received increasing attention. However, the development of KL injury is complex and polygenic, suggesting that a single SNP may not adequately capture an individual’s susceptibility. To examine whether total and weighted genotype scores are associated with KL injury incidence in adolescent basketball athletes, and whether these associations differ by sex.

**Methods:**

Data from 534 Japanese basketball athletes (participation age: 15–23 years) were retrospectively analyzed. KL injuries diagnosed between the ages of 13 and 17 years were recorded, and injury incidence was calculated per 1,000 athlete-hours (AHs). Eight SNPs in collagen- and matrix-related genes were genotyped. The total genotype score (TGS) was calculated by summing genotype scores. Weighted TGS (w-TGS) was calculated by weighting the genotype scores according to SNP-specific standardized regression coefficients. Associations between genotype scores and injury incidence were examined separately according to sex.

**Results:**

Female athletes demonstrated a higher incidence of KL injuries than male (*p* < 0.001). In females, w-TGS was positively associated with KL injury (*r* = 0.14, *p* = 0.01) incidence, whereas TGS was not. Female athletes with high w-TGS (0.15, 95% CI [0.08–0.22] AHs) exhibited a significantly greater KL injury incidence than females with low w-TGS (0.05, 95% CI [0.01–0.09] AHs, *p* = 0.014) and males regardless of w-TGS (HIGH in female vs. HIGH in male; 0.01, 95% CI [0.00-0.03] AHs, *p* < 0.001, vs. LOW in male; 0.01, 95% CI [0.00-0.03] AHs, *p* = 0.001). No significant association was observed among males. Among the individual SNPs, *COL5A1* rs13946 TT genotype was significantly associated with a higher incidence of KL injury in females (*p* = 0.005).

**Conclusions:**

These findings suggest that a w-TGS derived from a candidate gene approach may be associated with an increased incidence of KL injuries in adolescent female athletes. Accounting for SNP-specific effects may enhance the characterization of adolescent sex-specific genetic susceptibility to ligament injury. The current findings are exploratory and require validation in independent cohorts before application to risk prediction or individualized prevention.

**Supplementary Information:**

The online version contains supplementary material available at https://doi.org/10.1186/s13102-026-01884-6.

## Background

Knee ligament (KL) injuries and the number of related surgical procedures have increased in recent years, paralleling the growth in sports participation [1–3]. KL injury occurs frequently during adolescence (10–19 years old), and among these injuries, anterior cruciate ligament (ACL) injury is approximately 2–8-fold more common in females than in males [4–8]. ACL injury occurs via contact with another player or through non-contact mechanisms, which often occur during sudden stopping, landing from a jump, cutting, and rapid changes in direction [9, 10]. As basketball involves these high-risk movements, it is considered a sport with a high risk of KL injury [5, 10]. Notably, the incidence of ACL injury in female basketball athletes is three to four times higher than that in male athletes [11]. Although 69% of female athletes return to sports (RTS) after knee injuries, their RTS rate is lower than that of male athletes, and female athletes are more likely to discontinue sports participation [12–14]. Furthermore, knee injuries may lead to reduced athletic performance and shortened career duration after RTS [15, 16]. Therefore, elucidating the factors underlying sex differences in the incidence of KL injury among adolescent basketball athletes remains an important issue.

The intrinsic factors associated with KL injury include anatomical, neuromuscular, hormonal, and genetic factors [17, 18]. Among these intrinsic factors, genetic variations have received increasing attention, particularly single-nucleotide polymorphisms (SNPs) in genes encoding proteins involved in ligament structure and function [19, 20]. Ligaments predominantly comprises collagen, which accounts for approximately 75% of their dry weight, with type I collagen constituting the majority of collagen fibers [21, 22]. Types III, V, and XII collagen regulate the diameter of type I collagen fibrils and contribute to tissue stability and flexibility [23–26]. These collagen types are encoded by their respective genes (i.e. collagen I α1 (*COL1A1*), collagen III α1 (*COL3A1*), collagen V α1 (*COL5A1*), and collagen XII α1 (*COL12A1*) respectively produce the α1(I), α1(III), α1(V), and α1(XII) chains of type I, III, V, and XII collagen). Accordingly, SNPs in these collagen-related genes have been implicated in KL injury risk, including *COL1A1* rs1800012 (C > A) located in the first intron, *COL3A1* rs1800255 (G > A) in exon 30, *COL5A1* rs13946 and rs12722 (C > T) in the 3′-untranslated region (UTR), and *COL12A1* rs970547 (C > T) in exon 65 [20, 27–32]. In addition to collagen, proteoglycans such as decorin (DCN), aggrecan (ACAN), and elastic microfibril interface 1 (EMILIN1) play important roles in collagen fibrillogenesis, extracellular matrix organization, elastic tissue formation, and resistance to compressive mechanical loading [23, 33–35]. SNPs within the intronic regions of genes encoding these proteoglycans, including *DCN* rs516115 (T > C), *ACAN* rs1516797 (T > G), and *EMILIN1* rs2289360 (T > C), have also been associated with KL injury [36, 37]. Furthermore, variants in genes involved in extracellular matrix remodeling and angiogenesis are related to KL injury susceptibility [38, 39]. The rs679620 (T > C) polymorphism in exon 2 of the matrix metalloproteinase 3 (*MMP3*) gene, which plays a role in collagen degradation, has been associated with KL injury [38]. In addition, polymorphisms in the promoter region of the vascular endothelial growth factor A (*VEGFA*) gene, including rs699947 (A > C) and rs2010963 (C > G), are involved in the regulation of angiogenesis and have also been related to KL injury risk [39, 40]. These genetic variants may influence the structural integrity and mechanical properties of collagenous tissues, thereby increasing their susceptibility to sports-related KL injuries.

Most previous studies have examined the association between a single genetic difference and the risk of KL injury. However, the development of KL injury is complex and polygenic, suggesting that a single SNP may not adequately capture an individual’s susceptibility. To address this limitation, the total genotype score (TGS) was proposed to encode and sum multiple genetic polymorphisms using an algorithm developed by Williams and Folland, in which each polymorphism is scored as 2 (optimal), 1 (intermediate), or 0 (less optimal) [41]. This approach has been applied to sports injury research by Varillas-Delgado et al. [42], who reported that TGS demonstrates significant accuracy in discriminating muscle injured and non-injured athletes, whereas most individual SNPs fail to reveal significant differences [42]. In this approach, all genetic polymorphisms are assigned equal weights. Recently, Eynon et al. [43] emphasized that genetic polymorphisms may interact in a complex manner, suggesting that assigning equal weights to all variants may be inappropriate. Therefore, they proposed the use of a weighted TGS (w-TGS), in which individual polymorphisms are weighted according to their relative effect sizes [43]. Indeed, recent studies have demonstrated that w-TGS is associated with differences between non-athletes and athletes, as well as with interindividual variability in athletic performance [44–46]. While evidence remains limited and is still developing, individual differences in KL injury occurrence among athletes may be more clearly explained by considering multiple genotype scores related to ligament structures rather than by examining a single gene or SNP in isolation. However, whether TGS and w-TGS are associated with the risk of KL injury, or such associations differ by sex during adolescence remain unclear.

We hypothesized that athletes with higher TGS and w-TGS values, reflecting the combined effects of multiple high-risk genetic polymorphisms, would have a higher risk of KL injury. To test this hypothesis, this study aimed to examine the association between TGS and w-TGS and the incidence of KL injury in adolescent athletes using a candidate gene approach. The analyzed polymorphisms included *COL1A1* rs1800012, *COL3A1* rs1800255, *COL5A1* rs13946, *COL5A1* rs12722, *COL12A1* rs970547, *MMP3* rs679620, *DCN* rs516115, *EMILIN1* rs2289360, *ACAN* rs1516797, *VEGFA* rs699947, and *VEGFA* rs2010963. We further examined whether these associations differed by sex. Given the pronounced sex-related differences in biological maturation during adolescence and the relatively high incidence of KL injury during this developmental period, our analyses focused on injuries that occurred between the ages of 13 and 17 years [4, 47, 48].

## Methods

### Participants

This retrospective study included Japanese high-school and university basketball athletes recruited between June 2019 and March 2023. Eligible participants were aged 15–23 years and had been playing basketball regularly in training at least three times per week for ≥ 2 h per session for a minimum 3 years. Of the 565 athletes initially recruited, 26 were excluded because they did not meet the eligibility criteria and 5 were excluded because of incomplete data, resulting in a final sample of 534 athletes. Information concerning KL injuries between the ages of 13 and 17 years diagnosed by a physician, as well as anthropometric characteristics and training-related variables, were retrospectively collected using a questionnaire (Table 1). Orthopedic surgeons diagnosed KL injuries based on clinical examination and/or imaging findings. All athletes competed at the domestic competitive level, ranging from prefectural to national competitions. Written informed consent was obtained from all participants and, in the case of minors, from their legal guardians before enrollment. For minor participants, written informed assent was also obtained after providing information in an age-appropriate manner. This study protocol was approved by the Ethics Committee of Ritsumeikan University (BKC-LSMH-2021-007) and was conducted in accordance with the Declaration of Helsinki.

**Table 1:** Demographic and training characteristics of the basketball athletes (*n* = 534)

	Basketball athletes (*n* = 534)	Female (*n* = 334)	Male (*n* = 200)	*p* value
At time of participation (aged 15–23 years)				
Age (years)	17.6 ± 2.0	17.8 ± 2.0	17.1 ± 1.9	**< 0.001**
Height (cm)	168.8 ± 8.4	164.3 ± 6.4	174.6 ± 7.1	**< 0.001**
Weight (kg)	61.1 ± 9.0	56.9 ± 6.4	66.6 ± 8.8	**< 0.001**
BMI (kg/m^2^)	21.2 ± 1.9	20.9 ± 1.7	21.6 ± 2.0	**< 0.001**
Basketball experience (years)	8.4 ± 3.1	8.4 ± 3.0	8.5 ± 3.2	0.997
Competitive level (national/ regional or local), (%)	33.5/ 66.5	32.9/ 67.1	34.5/ 65.5	0.776
Between 13 and 17 years of age (retrospectively collected)				
Average basketball experience (years)	5.5 ± 2.1	5.7 ± 2.0	5.2 ± 2.2	**0.013**
Annual training time (hours)	986.8 ± 305.0	1,043.7 ± 323.2	914.3 ± 263.7	**< 0.001**
Training frequency (Number of days per week)	6.0 ± 0.6	6.0 ± 0.6	6.0 ± 0.5	0.745
Position (Guard/ Forward/ Center), (%)	40.1/ 40.7/ 19.2	43.8/ 38.5/ 17.7	34.0/ 44.3/ 21.7	0.140
Diagnosed by clinical examination with imaging (%)	85.7	84.6	100	1.000
Athletes with/without KL injury (%)	5.2/ 94.8	7.8/ 92.2	1.0/ 99.0	**< 0.001**
Athletes with/without ACL injury (%)	3.2/ 96.8	4.5/ 95.5	1.0/ 99.0	**0.038**
KL injury incidence (1,000AHs) ^a^	0.07 [0.04–0.10]	0.10 [0.06–0.15]	0.01 [0.00-0.02]	**< 0.001**
ACL injury incidence (1,000AHs) ^a^	0.04 [0.02–0.06]	0.06 [0.02–0.09]	0.01 [0.00-0.02]	**0.003**

Data are presented as mean ± SD unless otherwise stated (except competition level, position data, diagnosed by methods, and athletes with/without ligament injury). The *p* value represents the difference between Female vs. Male, and the value in bold indicates *p* < 0.05

*KL* knee ligament, *ACL* anterior cruciate ligament, *AHs* athlete-hours

^a^ Injury incidence is shown as means [95% confidence interval]

### Target gene polymorphisms

Using a candidate gene approach, we initially selected 11 SNPs that were previously reported to be associated with tendon and ligament injuries in athletes: *COL1A1* rs1800012, *COL3A1* rs1800255, *COL5A1* rs13946, *COL5A1* rs12722, *COL12A1* rs970547, *MMP3* rs679620, *DCN* rs516115, *EMILIN1* rs2289360, *ACAN* rs1516797, *VEGFA* rs699947, and *VEGFA* rs2010963 [49]. Polymorphisms with a minor allele homozygous frequency < 5% in the Japanese population, such as *COL1A1* rs1800012, *COL5A1* rs12722, and *EMILIN1* rs2289360, were excluded based on data from the Ensembl Genome Browser v.115 (https://asia.ensembl.org/index.html, accessed on December 5, 2025) [50]. Eight SNPs were included in the final analysis: *COL3A1* rs1800255, *COL5A1* rs13946, *COL12A1* rs970547, *MMP3* rs679620, *DCN* rs516115, *ACAN* rs1516797, *VEGFA* rs699947, and *VEGFA* rs2010963.

### Genotyping

Genomic DNA was extracted from buccal mucosa samples collected by vigorously rinsing the mouth with 10 mL of water for 1 min and collecting the samples in sterile tubes [51, 52]. Extracted DNA was diluted to a concentration of 10 ng/µL and stored at 4 °C until analysis. Genotyping was performed using TaqMan probes (Applied Biosystems (ABI), Foster City, CA, USA) and an ABI 7500 Fast real-time polymerase chain reaction (Real-Time PCR) platform (Life Technologies Japan, Tokyo, Japan). PCR conditions consisted of 60 °C for 1 min and 95 °C for 20 s (1 cycle), followed by 40 cycles of 95 °C for 3 s, 60 °C for 30 s, with a final extension step of 60 °C for 1 min [53]. Assay IDs for each SNP are provided Supplemental material (S1).

### Genotype scores

TGS was calculated using an algorithm proposed by Williams and Folland [41]. Each of the eight SNPs was assigned a score of 2 for “genotypes associated with higher injury risk,” 1 for “heterozygous genotypes associated with intermediate risk,” and 0 for " genotypes with a lower reported association with injury risk,” based on previous studies (Table 2) [29, 49, 54]. The summed genotype score was transformed into a 0-100 scale for ease of interpretation, with higher scores indicating greater injury risk. TGS was calculated as follows:

1 $$\begin{aligned} \mathrm{TGS}=&(\mathrm{GS}\;COL{\it3}A{\it1}+\mathrm{GS}\;COL{\it5}A{\it1}+\mathrm{GS}\;COL{\it12}A{\it1}\\&+\mathrm{GS}\;MMP{\it3}+\mathrm{GS}\;DCN+\mathrm{GS}\;ACAN\\&+\mathrm{GS}\;VEGFA\;699947+\mathrm{GS}\;VEGFA\;2010963)\\&\times100/16 \end{aligned}$$

**Table 2:** Candidate genetic polymorphisms for KL injury risk in athletes with each genotype score and weighted

Gene	rs Number	Genotype score	β-coefficient	Weighted- genotype score
*COL3A1*	rs1800255	AA = 2 GA = 1 GG = 0	-0.1	AA = -0.2 GA = -0.1 GG = 0
*COL5A1*	rs13946	TT = 2 CT = 1 CC = 0	3.9	TT = 7.8 CT = 3.9 CC = 0
*COL12A1*	rs970547	AA = 2 GA = 1 GG = 0	1.1	AA = 2.2 GA = 1.1 GG = 0
*MMP3*	rs679620	AA = 2 GA = 1 GG = 0	0.1	AA = 0.2 GA = 0.1 GG = 0
*DCN*	rs516115	AA = 2 GA = 1 GG = 0	0.2	AA = 0.4 GA = 0.2 GG = 0
*ACAN*	rs1516797	GG = 2 GT = 1 TT = 0	-0.2	GG = -0.4 GT = -0.2 TT = 0
*VEGFA*	rs699947	AA = 2 CA = 1 CC = 0	0.6	AA = 1.2 CA = 0.6 CC = 0
*VEGFA*	rs2010963	CC = 2 CG = 1 GG = 0	2.3	CC = 4.6 CG = 2.3 GG = 0

*COL3A1* collagen III α1, *COL5A1* collagen V α1, *COL12A1* collagen XII α1, *MMP3* matrix metalloproteinase 3, *DCN* decorin, *ACAN* aggrecan, *VEGFA* vascular endothelial growth factor A

To calculate w-TGS, standardized regression coefficients (β) for each SNP were obtained using multiple regression analysis following methods described by Varillas Delgado et al. [45] and McAuley et al. [46] (Table 2). This approach assumes that individual SNPs contribute differently to the risk of injury. The relative contribution of each SNP was calculated as follows:

2 $$\begin{aligned} &\text{SNP partial contribution}\\&=\left[\left(\beta\text{-coefficient for SNP}\right)/\sum\left(\text{of all }\beta\mathrm{-coefficient}\right)\right] \end{aligned}$$

Each SNP genotype score (2, 1, or 0) was multiplied by its corresponding partial contribution to obtain a weighted genotype score (w-GS). The w-GSs were then summed (w-TGS) and transformed to a 0-100 scale, with higher values indicating a greater injury risk, consistent with previous studies [45]. w-TGS was calculated as follows:

3 $$\begin{aligned} \mathrm{w-TGS}=&(\mathrm{w-GS}\;COL{\it3}A{\it1}+\mathrm{w-GS}\;COL{\it5}A{\it1}+\mathrm{w-GS}\;COL{\it12}A{\it1}\\&+\mathrm{w-GS}\;MMP{\it3}+\mathrm{w-GS}\;DCN+\mathrm{w-GS}\;ACAN\\&+\mathrm{w-GS}\;VEGFA\;699947+\mathrm{w-GS}\;VEGFA\;2010963)\\&\times100/16 \end{aligned}$$

### Incidence of KL injury

To account for differences in training time among athletes that might influence the risk of KL injury, KL injury incidence was calculated as the number of injuries per 1,000 h of athlete training time (1,000 athlete hours [AHs]) [55]. AHs were defined as one AH when one athlete participated in basketball training for one hour. The number of injuries that occurred during basketball training was divided by the annual training time to calculate the injury rate per 1,000 h. The accumulated training times between the ages of 13 and 17 years were summed to calculate the total AHs for each participant. The incidence of KL injuries was calculated using the following equation:

4 $$\text{Incidence rate}=\left(\text{number of injuries}/\text{training times}\right)\times1,000$$

### Statistical analysis

All statistical analyses were performed using SPSS Statistics (v.29.0; IBM Japan, Tokyo, Japan) and are expressed as means ± SD or means ± 95% confidence interval (95% CI). Hardy-Weinberg Equilibrium (HWE) for each SNP was assessed using the Chi-squared (χ^2^) test. Differences between sexes in participant characteristics were initially examined using χ^2^, Fisher’s exact test or unpaired Student’s t-tests. Participants were dichotomized into high- and low-genotype score groups based on respective TGS and w-TGS means. Specifically, participants with scores greater than the means were assigned to high-scoring groups (TGS HIGH and w-TGS HIGH), whereas those with scores equal to or lower than the means were assigned to lower-scoring groups (TGS LOW and w-TGS LOW). One-way analysis of variance (ANOVA) was performed separately for males and females to examine the effects of genotype score and sex on KL injury incidence. SNP-specific analyses were also conducted using one-way ANOVA stratified by sex. When significant main effects were detected, Tukey’s post hoc test was applied for multiple comparisons. In addition, analysis of covariance was performed with years of basketball experience as a covariate. Furthermore, associations between genotype scores (TGS and w-TGS) and the incidence of KL and ACL injuries were determined using Pearson’s correlation coefficient. Statistical significance was set at *p* < 0.05.

## Results

### Participant characteristics

Participant characteristics assessed at time of participation and retrospectively assessed between ages 13 and 17 years of age are summarized in Table 1. At time of participation, female athletes were significantly older, shorter, and lighter, and had lower BMI values than their male counterparts (*p* < 0.001), whereas basketball experience and competitive level at that time did not differ significantly by sex (Table 1). For the retrospectively assessed period between 13 and 17 years of age, basketball experience and annual training time were significantly greater among female athletes than male athletes (*p* = 0.013 and *p* < 0.001, respectively) (Table 1). No significant sex-based differences were observed in training frequency or playing position distribution (Table 1). KL injuries diagnosed during this period were confirmed by clinical examination with imaging in 84.6% of female athletes and in all male athletes (Table 1). Female athletes exhibited significantly higher incidences of both KL (*p* < 0.001) and ACL injuries (*p* = 0.003) compared to male athletes (Table 1).

**Fig. 1:**
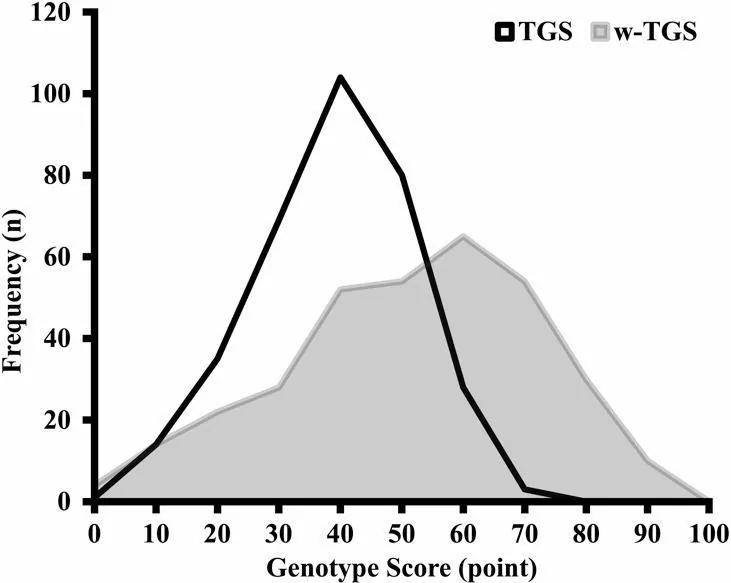
Distribution of total genotype scores (TGS) and weighted total genotype scores (W-TGS) in basketball athletes

All SNPs were in HWE, with no significant deviations observed in either female or male athletes (Table 3). The frequency distributions of TGS and w-TGS across the study population are shown in Fig. 1. Mean TGS and w-TGS for the total sample were 44.7 ± 11.2 and 56.5 ± 20.0, respectively. Sex-stratified values were as follows: TGS (females: 44.6 ± 11.4; males: 44.8 ± 11.0) and w-TGS (females: 55.9 ± 20.2; males: 57.2 ± 19.6). No significant difference in TGS or w-TGS was observed between female and male athletes. Participants were classified into HIGH and LOW groups based on TGS and w-TGS means. TGS was 55.5 ± 6.3 in the HIGH_Female group, 54.7 ± 5.2 in the HIGH_Male group, 37.0 ± 7.1 in the LOW_Female group, and 37.2 ± 7.7 in the LOW_Male group. Corresponding w-TGS were 71.6 ± 9.5 in the HIGH_Female group, 73.0 ± 9.6 in the HIGH_Male group, 38.4 ± 13.3 in the LOW_Female group, and 40.1 ± 11.8 in the LOW_Male group. Within both HIGH and LOW groups, no significant difference in TGS or w-TGS was observed between female and male athletes.

**Table 3:** Genotypic distribution of polymorphisms studied among male and female basketball athletes

SNPs	Genotype	Female athlete *n* (%)	Male athlete *n* (%)	Ensemble genome %	HWE *p* value
*COL3A1* rs1800255	AA GA GG	33 (9.9) 138 (41.3) 163 (48.8)	12 (6.0) 77 (38.5) 111 (55.5)	5.8 43.3 51.0	0.17
*COL5A1* rs13946	TT CT CC	144 (43.1) 145 (43.4) 45 (13.5)	86 (43.0) 91 (45.5) 23 (11.5)	34.6 54.8 10.6	0.78
*COL12A1* rs970547	AA GA GG	140 (41.9) 153 (45.8) 41 (12.3)	93 (46.5) 83 (41.5) 24 (12.0)	48.1 41.3 10.6	0.57
*MMP3* rs679620	AA GA GG	40 (12.0) 137 (41.0) 157 (47.0)	27 (13.5) 83 (41.5) 90 (45.0)	7.7 41.3 51.0	0.84
*DCN* rs516115	AA GA GG	131 (39.2) 154 (46.1) 49 (14.7)	79 (39.5) 98 (49.0) 23 (11.5)	38.5 41.3 20.2	0.56
*ACAN* rs1516797	GG GT TT	20 (6.0) 148 (44.3) 166 (49.7)	15 (7.5) 75 (37.5) 110 (55.0)	6.7 50.0 43.3	0.29
*VEGFA* rs699947	AA CA CC	28 (8.4) 152 (45.5) 154 (46.1)	25 (12.5) 79 (39.5) 96 (48.0)	11.5 44.2 44.2	0.19
*VEGFA* rs2010963	CC CG GG	63 (18.9) 156 (46.7) 115 (34.4)	34 (17.0) 105 (52.5) 61 (30.5)	13.5 50.0 36.5	0.43

*HWE* Hardy–Weinberg Equilibrium, *COL3A1* collagen III α1, *COL5A1* collagen V α1, *COL12A1* collagen XII α1, *MMP3* matrix metalloproteinase 3, *DCN* decorin, *ACAN* aggrecan, *VEGFA* vascular endothelial growth factor A

Among female athletes, w-TGS associated positively with KL incidence (*r* = 0.14, *p* = 0.01) and ACL injuries (*r* = 0.12, *p* = 0.04). No significant association was observed between TGS and incidence of injury. Simple linear regression confirmed these associations, with w-TGS accounting for approximately 2% of the variance in KL injury incidence (*R*² = 0.020, *F* = 6.793, *p* = 0.010) and 1% of the variance in ACL injury incidence (*R*² = 0.013, *F* = 4.477, *p* = 0.035). No significant association was identified between genotype scores and injury incidence in male athletes.

### Effects of genotype scores and SNPs on KL injury incidence

**Fig. 2:**
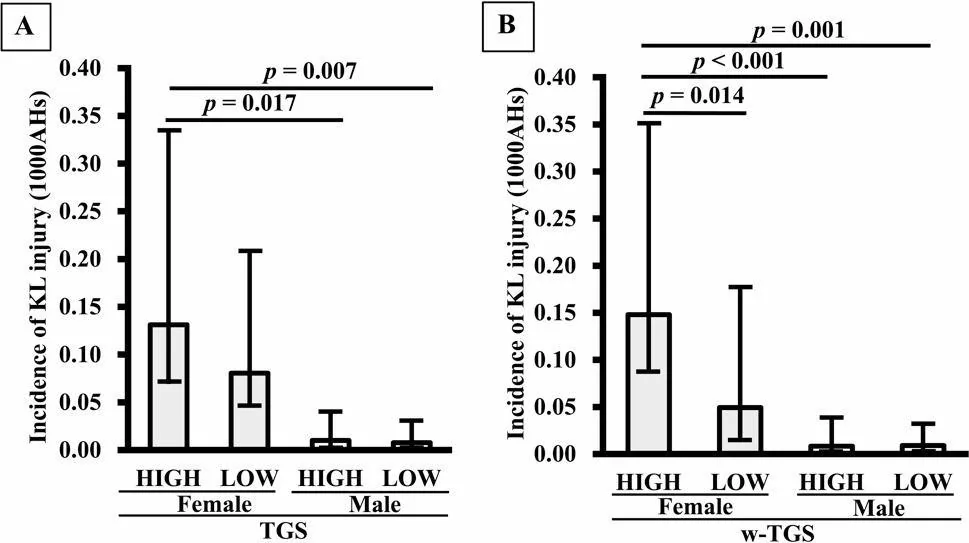
Sex-stratified KL injury incidence by total and weighted total genotype score (TGS (**A**) and w-TGS(**B**))

Respective KL injury incidence data from TGS and w-TGS HIGH and LOW groups and stratified by sex is shown in Fig. 2A and B. The incidence of KL injury was higher in the TGS HIGH_Female group than in the TGS HIGH_Male (*p* = 0.017) and TGS LOW_Male groups (*p* = 0.007) (Fig. 2A). However, no significant differences were observed between the TGS HIGH_Female and TGS LOW_Female groups (Fig. 2A). In the w-TGS HIGH_Female group, the incidence of KL injury was significantly higher than that in both the w-TGS HIGH_Male (*p* < 0.001) and w-TGS LOW_Male groups (*p* = 0.001) (Fig. 2B). Furthermore, the w-TGS HIGH_Female group demonstrated a significantly higher incidence of KL injury than the w-TGS LOW_Female group (*p* = 0.014) (Fig. 2B).

Among individual SNPs, female athletes with the TT genotype of *COL5A1* rs13946 showed a significantly higher incidence of KL injury than in those with the CC + CT genotype (*p* = 0.005) (Table 4). No significant associations were observed for the other SNPs in female athletes. In male athletes, no significant associations were observed with any of the SNPs. Across all SNPs analyzed, KL injury incidence was higher among female athletes than male athletes (*p* < 0.05) (Supplemental material (S2)).

**Table 4:** Association between each SNP and KL injury incidence by sex in basketball athletes

	Female athletes *n* = 334					Male athletes *n* = 200				
SNPs	Genotype (n)		Incidence rate (1,000AHs [95% CI])	*p*	p*	Genotype (n)		Incidence rate (1,000AHs [95% CI])	*p*	p*
*COL3A1* rs1800255	AA (33) vs. GA + GG (301)	AA GA + GG	0.06 [0.00–0.15] 0.11 [0.06–0.15]	0.82	0.95	AA (12) vs. GA + GG (188)	AA GG + GA	0.00 [0.00–0.00] 0.01 [0.00–0.02]	1.00	1.00
	AA + GA (171) vs. GG (163)	AA + GA GG	0.10 [0.04–0.15] 0.10 [0.05–0.16]	1.00	1.00	AA + GA (89) vs. GG (111)	AA + GA GG	0.01 [0.00–0.03] 0.01 [0.00–0.02]	1.00	1.00
*COL5A1* rs13946	TT (144) vs. CT + CC (190)	TT CT + CC	0.16 [0.08–0.24] 0.05 [0.02–0.09]	**0.005**	0.72	TT (86) vs. CT + CC (114)	TT CT + CC	0.00 [0.00–0.00] 0.02 [0.00–0.04]	1.00	1.00
	TT + CT (289) vs. CC (45)	TT + CT CC	0.11 [0.06–0.15] 0.06 [0.00–0.16]	0.81	1.00	TT + CT (177) vs. CC (23)	TT + CT CC	0.01 [0.00–0.02] 0.00 [0.00–0.00]	1.00	1.00
*COL12A1* rs970547	AA (140) vs. GA + GG (194)	AA GA + GG	0.12 [0.05–0.19] 0.09 [0.04–0.14]	0.79	0.34	AA (93) vs. GA + GG (107)	AA GA + GG	0.00 [0.00–0.00] 0.02 [0.00–0.04]	0.98	1.00
	AA + GA (293) vs. GG (41)	AA + GA GG	0.11 [0.07–0.15] 0.06 [0.00–0.17]	0.74	0.88	AA + GA (176) vs. GG (24)	AA + GA GG	0.01 [0.00–0.02] 0.00 [0.00–0.00]	1.00	1.00
*MMP3* rs679620	AA (40) vs. GA + GG (294)	AA GA + GG	0.10 [0.00–0.23] 0.10 [0.06–0.14]	1.00	1.00	AA (27) vs. GA + GG (173)	AA GA + GG	0.00 [0.00–0.00] 0.01 [0.00–0.02]	1.00	1.00
	AA + GA (177) vs. GG (157)	AA + GA GG	0.11 [0.05–0.16] 0.10 [0.04–0.15]	1.00	1.00	AA + GA (110) vs. GG (90)	AA + GA GG	0.01 [0.00–0.02] 0.01 [0.00–0.03]	1.00	1.00
*DCN* rs516115	AA (131) vs. GA + GG (203)	AA GA + GG	0.10 [0.04–0.17] 0.10 [0.05–0.15]	1.00	1.00	AA (79) vs. GA + GG (121)	AA GA + GG	0.01 [0.00–0.03] 0.01 [0.00–0.02]	1.00	1.00
	AA + GA (285) vs. GG (49)	AA + GA GG	0.10 [0.06–0.15] 0.09 [0.00–0.17]	1.00	1.00	AA + GA (177) vs. GG (23)	AA + GA GG	0.01 [0.00–0.02] 0.00 [0.00–0.00]	1.00	1.00
*ACAN* rs1516797	GG (20) vs. GT + TT (314)	GG GT + TT	0.11 [0.00–0.35] 0.10 [0.06–0.14]	1.00	0.99	GG (15) vs. GT + TT (185)	GG GT + TT	0.00 [0.00–0.00] 0.01 [0.00–0.02]	1.00	1.00
	GG + GT (168) vs. TT (166)	GG + GT TT	0.10 [0.04–0.15] 0.11 [0.05–0.16]	0.99	0.97	GG + GT (90) vs. TT (110)	GG + GT TT	0.01 [0.00–0.03] 0.01 [0.00–0.02]	1.00	1.00
*VEGFA* rs699947	AA (28) vs. CA + CC (306)	AA CA + CC	0.04 [0.00–0.13] 0.11 [0.06–0.15]	0.69	1.00	AA (25) vs. CA + CC (175)	AA CA + CC	0.00 [0.00–0.00] 0.01 [0.00–0.02]	1.00	1.00
	AA + CA (180) vs. CC (154)	AA + CA CC	0.11 [0.05–0.17] 0.09 [0.04–0.14]	0.88	1.00	AA + CA (104) vs. CC (96)	AA + CA CC	0.00 [0.00–0.00] 0.02 [0.00–0.04]	0.97	1.00
*VEGFA* rs2010963	CC (63) vs. CG + GG (271)	CC CG + GG	0.09 [0.01–0.16] 0.11 [0.06–0.15]	0.96	1.00	CC (34) vs. CG + GG (166)	CC CG + GG	0.03 [0.00–0.08] 0.01 [0.00–0.02]	0.98	1.00
	CC + CG (219) vs. GG (115)	CC + CG GG	0.13 [0.07–0.18] 0.06 [0.00–0.11]	0.17	1.00	CC + CG (139) vs. GG (61)	CC + CG GG	0.01 [0.00–0.03] 0.00 [0.00–0.00]	0.99	1.00

Value in bold indicate *p* < 0.05

*COL3A1* collagen III α1, *COL5A1* collagen V α1, *COL12A1* collagen XII α1, *MMP3* matrix metalloproteinase 3, *DCN* decorin, *ACAN* aggrecan, *VEGFA* vascular endothelial growth factor A

*p value adjusted for basketball experience years

After adjusting for basketball experience years as a covariate, the group differences in KL injury incidence observed for both TGS and w-TGS remained statistically significant (*p* < 0.05). No significant differences in SNP-specific injury incidence were observed following covariate adjustment. These data are provided in Table 4 and Supplemental material (S2).

### Effects of genotype scores and SNPs on ACL injury incidence

**Fig. 3:**
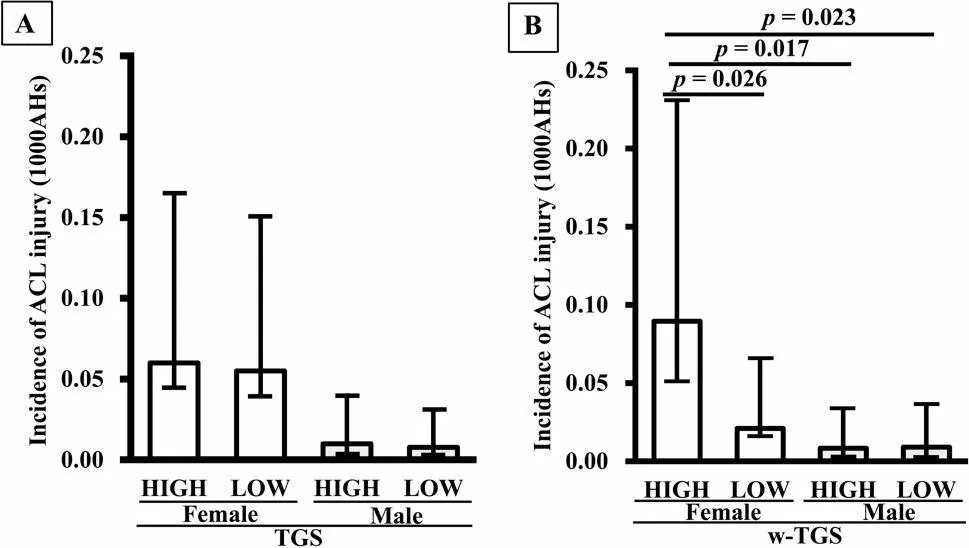
Sex-stratified ACL injury incidence by Total and weighted total genotype score (TGS (**A**) and w-TGS(**B**))

Respective ACL injury incidences across TGS and w-TGS HIGH and LOW groups stratified by sex are presented in Fig. 3A and B. The incidence of ACL injury was not significantly different between the TGS groups (Fig. 3A). In the analysis of w-TGS and ACL injury incidence, the w-TGS HIGH_Female group exhibited a significantly higher incidence of ACL injury than the w-TGS HIGH_Male (*p* = 0.017) and w-TGS LOW_Male groups (*p* = 0.023) (Fig. 3B). Furthermore, the w-TGS HIGH_Female group showed a significantly higher incidence than the w-TGS LOW_Female group (*p* = 0.026) (Fig. 3B).

Regarding the effects of individual SNPs, the incidence of ACL injury was significantly higher in female athletes with the TT genotype of *COL5A1* rs13946 than in those with the CC + CT genotype (*p* = 0.006) (Table 5). Additionally, female and male athletes were compared, female athletes with the TT genotype of *COL5A1* rs13946 exhibited a significantly higher incidence of ACL injury than male athletes with the TT genotype (*p* = 0.004) (Supplemental material (S2)). No significant associations were observed between the other SNPs and ACL injury incidence in female athletes, and no SNPs were significantly associated with ACL injury incidence in male athletes (Table 5 and Supplemental material (S2)).

**Table 5:** Association between each SNP and ACL injury incidence by sex in basketball athletes

	Female athletes *n* = 334					Male athletes *n* = 200				
SNPs	Genotype (n)		Incidence rate (1,000AHs [95% CI])	*p*	p*	Genotype (n)		Incidence rate (1,000AHs [95% CI])	*p*	p*
*COL3A1* rs1800255	AA (33) vs. GA + GG (301)	AA GA + GG	0.03 [0.00–0.09] 0.06 [0.03–0.09]	0.89	1.00	AA (12) vs. GA + GG (188)	AA GG + GA	0.00 [0.00–0.00] 0.01 [0.00–0.02]	1.00	1.00
	AA + GA (171) vs. GG (163)	AA + GA GG	0.04 [0.01–0.08] 0.07 [0.02–0.12]	0.61	0.97	AA + GA (89) vs. GG (111)	AA + GA GG	0.01 [0.00–0.03] 0.01 [0.00–0.02]	1.00	1.00
*COL5A1* rs13946	TT (144) vs. CT + CC (190)	TT CT + CC	0.10 [0.04–0.16] 0.02 [0.00–0.05]	**0.006**	0.34	TT (86) vs. CT + CC (114)	TT CT + CC	0.00 [0.00–0.00] 0.02 [0.00–0.04]	0.96	1.00
	TT + CT (289) vs. CC (45)	TT + CT CC	0.06 [0.03–0.10] 0.02 [0.00–0.07]	0.67	0.96	TT + CT (177) vs. CC (23)	TT + CT CC	0.01 [0.00–0.02] 0.00 [0.00–0.00]	1.00	1.00
*COL12A1* rs970547	AA (140) vs. GA + GG (194)	AA GA + GG	0.06 [0.02–0.10] 0.06 [0.02–0.10]	1.00	1.00	AA (93) vs. GA + GG (107)	AA GA + GG	0.00 [0.00–0.00] 0.02 [0.00–0.04]	0.96	1.00
	AA + GA (293) vs. GG (41)	AA + GA GG	0.06 [0.03–0.09] 0.06 [0.00–0.17]	1.00	1.00	AA + GA (176) vs. GG (24)	AA + GA GG	0.01 [0.00–0.02] 0.00 [0.00–0.00]	1.00	1.00
*MMP3* rs679620	AA (40) vs. GA + GG (294)	AA GA + GG	0.00 [0.00–0.00] 0.07 [0.03–0.10]	0.31	0.87	AA (27) vs. GA + GG (173)	AA GA + GG	0.00 [0.00–0.00] 0.01 [0.00–0.02]	1.00	1.00
	AA + GA (177) vs. GG (157)	AA + GA GG	0.05 [0.02–0.09] 0.06 [0.02–0.11]	0.94	1.00	AA + GA (110) vs. GG (90)	AA + GA GG	0.01 [0.00–0.02] 0.01 [0.00–0.03]	1.00	1.00
*DCN* rs516115	AA (131) vs. GA + GG (203)	AA GA + GG	0.06 [0.02–0.11] 0.05 [0.02–0.09]	0.99	1.00	AA (79) vs. GA + GG (121)	AA GA + GG	0.01 [0.00–0.03] 0.01 [0.00–0.02]	1.00	1.00
	AA + GA (285) vs. GG (49)	AA + GA GG	0.06 [0.02–0.09] 0.07 [0.00–0.14]	0.99	1.00	AA + GA (177) vs. GG (23)	AA + GA GG	0.01 [0.00–0.02] 0.00 [0.00–0.00]	1.00	1.00
*ACAN* rs1516797	GG (20) vs. GT + TT (314)	GG GT + TT	0.06 [0.00–0.18] 0.06 [0.03–0.09]	1.00	1.00	GG (15) vs. GT + TT (185)	GG GT + TT	0.00 [0.00–0.00] 0.01 [0.00–0.02]	1.00	1.00
	GG + GT (168) vs. TT (166)	GG + GT TT	0.05 [0.01–0.10] 0.06 [0.02–0.10]	0.99	1.00	GG + GT (90) vs. TT (110)	GG + GT TT	0.01 [0.00–0.03] 0.01 [0.00–0.02]	1.00	1.00
*VEGFA* rs699947	AA (28) vs. CA + CC (306)	AA CA + CC	0.04 [0.00–0.13] 0.06 [0.03–0.09]	0.98	1.00	AA (25) vs. CA + CC (175)	AA CA + CC	0.00 [0.00–0.00] 0.01 [0.00–0.02]	1.00	1.00
	AA + CA (180) vs. CC (154)	AA + CA CC	0.06 [0.02–0.10] 0.05 [0.01–0.10]	1.00	1.00	AA + CA (104) vs. CC (96)	AA + CA CC	0.00 [0.00–0.00] 0.02 [0.00–0.04]	0.94	1.00
*VEGFA* rs2010963	CC (63) vs. CG + GG (271)	CC CG + GG	0.04 [0.00–0.10] 0.06 [0.03–0.09]	0.93	1.00	CC (34) vs. CG + GG (166)	CC CG + GG	0.03 [0.00–0.08] 0.01 [0.00–0.02]	0.96	1.00
	CC + CG (219) vs. GG (115)	CC + CG GG	0.07 [0.03–0.11] 0.04 [0.00–0.08]	0.74	1.00	CC + CG (139) vs. GG (61)	CC + CG GG	0.01 [0.00–0.03] 0.00 [0.00–0.00]	0.98	1.00

Value in bold indicate *p* < 0.05

*COL3A1* collagen III α1, *COL5A1* collagen V α1, *COL12A1* collagen XII α1, *MMP3* matrix metalloproteinase 3, *DCN* decorin, *ACAN* aggrecan, *VEGFA* vascular endothelial growth factor A

*p value adjusted for basketball experience years

After adjusting for basketball experience years as a covariate, the w-TGS HIGH_Female group displayed the highest ACL injury incidence among all groups (*p* < 0.05). However, no significant differences in incidence were observed for any of the SNPs. These data are provided in Table 5 and Supplemental material (S2).

## Discussion

This study investigated the effect of combining eight genetic polymorphisms in genotype scores, using a candidate gene approach, on the incidence of KL injury and sex differences in adolescent Japanese basketball athletes. The main findings indicated that female athletes with high w-TGS scores had a higher incidence of KL injury than female athletes with low w-TGS scores and male athletes. In contrast, no differences in the KL injury incidence were observed among male athletes based on either weighted or unweighted genotype scores. Additionally, in female athletes, the incidence of KL injury increased with higher w-TGS. These results suggest that sex differences exist in genetic factors associated with the risk of adolescent KL injuries among Japanese basketball athletes.

This study demonstrated that a higher w-TGS was associated with an increased risk of KL injury in adolescent female athletes, whereas TGS was not associated with injury risk. Although TGS has been suggested to be useful in relation to sports performance and muscle injury, this study was unable to confirm its association with KL injury [42, 56, 57]. Eynon et al. [43] demonstrated that the effectiveness of the genotype scoring approach depends on factors such as the selection of appropriate polymorphisms and the weighting applied to each genotype. In fact, Massidda et al. [44] showed that w-TGS explained a higher proportion of the variance in vertical jump performance than TGS. Similarly, McAuley et al. [46] demonstrated a higher explanatory power for the w-TGS than for TGS in soccer skills, including dribbling, passing, and shooting. In this study, simple linear regression analysis indicated that w-TGS explained approximately 2% of the variance in KL injury incidence (*R²* = 0.020, *p* = 0.010), suggesting that weighting individual SNPs according to their effect sizes may enhance their ability to detect genetic associations, although overall explanatory power was modest. This finding is consistent with previous studies [46, 58]. Furthermore, the results of this study suggested that the influence of genotype scores on the incidence of KL injury may differ according to sex. The eight SNPs selected in this study appeared to exert different levels of influence, and a genotype score accounting for these differences was associated with the incidence of KL injury. These findings suggest that w-TGS may be a useful indicator of genetic associations with KL injury incidence, particularly among adolescent female athletes. Replication in independent cohorts is needed before clinical application can be considered.

In the present study, SNP analysis revealed that only the TT genotype of *COL5A1* rs13946 was associated with the incidence of KL injury in female basketball athletes. *COL5A1* rs13946 consistently exhibited the largest standardized regression coefficient among the included SNPs, resulting in the greatest contribution to w-TGS in this study. Previous studies have reported an association between the TT genotype of *COL5A1* rs13946 and KL injury risk, supporting the present findings [59, 60]. Located in the 3′-UTR region, this SNP has been suggested to influence mRNA stability, potentially altering type V collagen expression and fibrillogenesis [61]. Additionally, the *VEGFA* rs2010963 polymorphism exhibited the second-greatest contribution to w-TGS, although no statistically significant association was observed in SNP-specific analysis. The *VEGFA* rs2010963 CC genotype was associated with ACL injury in male soccer athletes before maturity, increasing the risk of ligament injury 10.3-fold compared with the GG genotype [39]. The *VEGFA* rs2010963 polymorphism may increase serum VEGFA levels and enhance MMP3 mRNA and protein expression, which could influence ligament biomechanical strength [62–65]. Similarly, although no significant association was observed between *COL12A1* rs970547, *MMP3* rs679620, DCN rs516115, and *VEGFA* rs699947, these polymorphisms contributed modestly to w-TGS. These findings suggest that partial w-TGS derived from multiple SNPs may have a genetic influence on the incidence of KL injury in female basketball athletes. Interestingly, *COL3A1* rs1800255 and *ACAN* rs1516797 were assigned negative weightings in this study, which contrasts with findings reported in Caucasian athletes and physically active individuals [29, 36, 66]. This discrepancy suggests that genetic influences on KL injury risk differ according to ethnic background [67]. Therefore, ethnic differences should be considered when interpreting genetic associations with KL injury risk. Thus, combining multiple genetic polymorphisms and accounting for their relative contributions may improve the detection of genetic influences on susceptibility to KL injury.

In the present study, no significant associations were detected in male athletes. These findings are based on sex-stratified analyses rather than a formal test of a sex-by-genotype score interaction effect. Therefore, while the present findings indicate that genotype scores associate with KL injury incidence in female athletes but not in male athletes, the following explanations for this sex-specific finding should be regarded as post hoc interpretations rather than confirmed mechanisms. One plausible explanation is the substantially lower incidence of KL and ACL injuries in males than in females in the present cohort, which may limit the statistical power for detecting genotype-related differences. Additionally, sex-specific differences in biological maturation during adolescence, including the hormonal milieu, neuromuscular control, and joint laxity, may modulate the extent to which genetic susceptibility is expressed. Finally, genetic influences on injury risk may interact with exposure-related factors, and such gene-environment interactions may differ by sex. Future studies incorporating formal interaction testing and larger male athlete cohorts are needed to clarify whether the observed sex differences in genotype score associations reflect true biological differences or are attributable to differences in statistical power.

This study had certain limitations. First, the sample size was limited to high-school and university athletes who participated in basketball. Although the statistical power was 76%, these findings should be interpreted with some caution. Nevertheless, the sample sizes were larger than those of previous cross-sectional studies involving athletes from the same sports and genotype score analyses [39, 44–46]. Second, the weighting of the SNPs was derived from multiple regression analyses within the same cohort. The present findings, particularly those related to sex-specific associations, should thus be considered exploratory. The possibility of overfitting when interpreting the w-TGS-based results cannot be excluded. The observed associations may be specific to this population and may not be generalizable to other cohorts of Japanese athletes. Finally, we employed a candidate gene approach rather than a genome-wide association study (GWAS). GWAS may identify additional polymorphisms associated with ligament injuries and uncover the effects of unexamined genetic variants. Therefore, replication in a larger, independent cohort of Japanese basketball athletes is required before these findings can be applied in practice. Collectively, the present findings suggest that combined and weighted candidate gene scores may provide a basis for characterizing genetic susceptibility to KL injury during adolescence and may contribute to the understanding of sex differences in injury risk.

## Conclusions

These findings suggest that selected genotype scores derived from a candidate gene approach, particularly when weighted for SNP-specific effects, may be associated with sex differences in adolescent KL injury incidence among Japanese basketball athletes. Genetic susceptibility to KL injury may be more comprehensively characterized by combining multiple SNPs and accounting for their relative contributions. These findings are exploratory in nature, and the observed associations should be interpreted within the context of the present cross-sectional design and cohort-specific weighting approach. As additional SNPs associated with KL injury and ethnic differences are identified, the reproducibility and predictive accuracy of genotype scores may improve. At present, any potential improvement in predictive utility remains hypothetical and would require validation in independent cohorts before genotype scores can be considered for risk stratification.

## Supplementary Information

Supplementary Material 1.

Supplementary Material 2.

## Data Availability

A subset of the dataset supporting the conclusions of this article, including individual genotypes, age, and sex, is available in the Figshare repository at https://doi.org/10.6084/m9.figshare.32144488. Other clinical data, such as injury incidence, are not publicly available to protect the privacy of the participants (minor athletes) and prevent potential re-identification. However, these data presented in this study are available from the corresponding author upon reasonable request.

## Abbreviations

ACAN: Aggrecan
ACL: Anterior cruciate ligament
AHs: Athlete-hours
ANOVA: One-way analysis of variance
COL1A1: Collagen I α 1
COL3A1: Collagen III α 1
COL5A1: Collagen V α 1
COL12A1: Collagen XII α 1
DCN: Decorin
EMILIN-1: Elastic microfibril interface 1
HWE: Hardy-Weinberg Equilibrium
KL: Knee ligament
MMP3: Matrix metalloproteinase 3
RTS: Return to sports
SNPs: Single-nucleotide polymorphisms
TGS: Total genotype score
VEGFA: Vascular endothelial growth factor A
w-TGS: weighted Total Genotype Score
95% CI: 95% Confidence interval
