# Supplementary material for: Weighted total genotype score associated with knee ligament injury incidence in adolescent female basketball athletes
Source: BMC Sports Sci Med Rehabil. 2026 Jul 14;18:472. doi: 10.1186/s13102-026-01884-6 (PMC13651358; doi:10.1186/s13102-026-01884-6)
Supplement: Supplementary file 1 [file 13102_2026_1884_MOESM1_ESM.docx]

**Supplemental material (S1). Assay IDs of the TaqMan probes used to determine gene polymorphisms in this study (Applied Biosystems, Foster City, CA, USA).**

| SNPs | Genotype | Assay ID |
| --- | --- | --- |
| COL3A1 rs1800255 | G → A | C___7477926_10 |
| COL5A1 rs13946 | C → T | C___8721600_10 |
| COL12A1 rs970547 | C → T | C___7580617_10 |
| MMP3 rs679620 | G → A | C___3047717_1_ |
| DCN rs516115 | C → T | C___2309580_10 |
| ACAN rs1516797 | T → G | C____331789_10 |
| VEGFA rs699947 | C → A | C___8311602_10 |
| VEGFA rs2010963 | G → C | C___8311614_10 |

COL3A1, collagen 3 alpha 1, COL5A1, collagen 5 alpha 1, COL12A1, collagen 12 alpha 1, MMP3, matrix metalloproteinase 3, DCN, decorin, ACAN, aggrecan, VEGFA, vascular endothelial growth factor A
