# Supplementary material for: Weighted total genotype score associated with knee ligament injury incidence in adolescent female basketball athletes
Source: BMC Sports Sci Med Rehabil. 2026 Jul 14;18:472. doi: 10.1186/s13102-026-01884-6 (PMC13651358; doi:10.1186/s13102-026-01884-6)
Supplement: Supplementary file 2 [file 13102_2026_1884_MOESM2_ESM.docx]

**Supplemental material (S2). KL and ACL injury incidence by individual SNP and sex difference in basketball athletes.**

|  |  | | KL injury | | | ACL injury | | |
| --- | --- | --- | --- | --- | --- | --- | --- | --- |
| SNPs | Genotype | Female / Male (n) | Incidence rate (1,000AHs [95% CI])  Female/ Male | *p* | *p** | Incidence rate (1,000AHs [95% CI])  Female/ Male | *p* | *p** |
| *COL3A1* rs1800255 | AA  GA+GG | 33 /12  301/188 | 0.06 [0.00-0.15]/ 0.00 [0.00-0.00]  0.11 [0.06-0.15]/ 0.01 [0.00-0.02] | 0.94  **0.003** | 1.00  0.18 | 0.03 [0.00-0.09]/ 0.00 [0.00-0.00]  0.06 [0.03-0.09]/ 0.01 [0.00-0.02] | 0.98  0.07 | 1.00  0.85 |
|  | AA+GA  GG | 171/ 89  163/ 111 | 0.10 [0.04-0.15]/ 0.01 [0.00-0.03]  0.10 [0.05-0.16]/ 0.01 [0.00-0.02] | 0.11  **0.047** | 0.81  0.72 | 0.04 [0.01-0.08]/ 0.01 [0.00-0.03]  0.07 [0.02-0.12]/ 0.01 [0.00-0.02] | 0.68  0.09 | 1.00  0.72 |
| *COL5A1* rs13946 | TT  CT+CC | 144/ 86  190/ 114 | 0.16 [0.08-0.24]/ 0.00 [0.00-0.00]  0.05 [0.02-0.09]/ 0.02 [0.00-0.04] | **< 0.001**  0.68 | 0.30  0.97 | 0.10 [0.04-0.16]/ 0.00 [0.00-0.00]  0.02 [0.00-0.05]/ 0.02 [0.00-0.04] | **0.004**  0.99 | 0.42  1.00 |
|  | TT+CT  CC | 289/ 177  45/ 23 | 0.11 [0.06-0.15]/ 0.01 [0.00-0.02]  0.06 [0.00-0.16]/ 0.00 [0.00-0.00] | **0.004**  0.84 | 0.49  0.97 | 0.06 [0.03-0.10]/ 0.01 [0.00-0.02]  0.02 [0.00-0.07]/ 0.00 [0.00-0.00] | 0.07  0.98 | 0.76  1.00 |
| *COL12A1* rs970547 | AA  GA+GG | 140/ 93  194/ 107 | 0.12 [0.05-0.19]/ 0.00 [0.00-0.00]  0.09 [0.04-0.14]/ 0.02 [0.00-0.04] | **0.016**  0.19 | 0.24  0.99 | 0.06 [0.02-0.10]/ 0.00 [0.00-0.00]  0.06 [0.02-0.10]/ 0.02 [0.00-0.04] | 0.23  0.42 | 0.97  0.99 |
|  | AA+GA  GG | 293/ 176  41/ 24 | 0.11 [0.07-0.15]/ 0.01 [0.00-0.02]  0.06 [0.00-0.17]/ 0.00 [0.00-0.00] | **0.004**  0.88 | 0.23  1.00 | 0.06 [0.03-0.09]/ 0.01 [0.00-0.02]  0.06 [0.00-0.17]/ 0.00 [0.00-0.00] | 0.12  0.76 | 0.92  1.00 |
| *MMP3* rs679620 | AA  GA+GG | 40/ 27  294/ 173 | 0.10 [0.00-0.23]/ 0.00 [0.00-0.00]  0.10 [0.06-0.14]/ 0.01 [0.00-0.02] | 0.54  **0.008** | 0.98  0.59 | 0.00 [0.00-0.00]/ 0.00 [0.00-0.00]  0.07 [0.03-0.10]/ 0.01 [0.00-0.02] | 1.00  0.05 | 1.00  0.79 |
|  | AA+GA  GG | 177/ 110  157/ 90 | 0.11 [0.05-0.16]/ 0.01 [0.00-0.02]  0.10 [0.04-0.15]/ 0.01 [0.00-0.03] | 0.13  **0.036** | 0.67  0.95 | 0.05 [0.02-0.09]/ 0.01 [0.00-0.02]  0.06 [0.02-0.11]/ 0.01 [0.00-0.03] | 0.40  0.25 | 0.98  0.98 |
| *DCN* rs516115 | AA  GA+GG | 131/ 79  203/ 121 | 0.10 [0.04-0.17]/ 0.01 [0.00-0.03]  0.10 [0.05-0.15]/ 0.01 [0.00-0.02] | 0.13  **0.038** | 0.88  0.76 | 0.06 [0.02-0.11]/ 0.01 [0.00-0.03]  0.05 [0.02-0.09]/ 0.01 [0.00-0.02] | 0.37  0.27 | 0.99  0.97 |
|  | AA+GA  GG | 285/ 171  49/ 23 | 0.10 [0.06-0.15]/ 0.01 [0.00-0.02]  0.09 [0.00-0.17]/ 0.00 [0.00-0.00] | **0.006**  0.65 | 0.51  0.98 | 0.06 [0.02-0.09]/ 0.01 [0.00-0.02]  0.07 [0.00-0.14]/ 0.00 [0.00-0.00] | 0.15  0.63 | 0.98  0.98 |
| *ACAN* rs1516797 | GG  GT+TT | 20/ 15  314/ 185 | 0.11 [0.00-0.35]/ 0.00 [0.00-0.00]  0.10 [0.06-0.14]/ 0.01 [0.00-0.02] | 0.69  **0.006** | 1.00  0.16 | 0.06 [0.00-0.18]/ 0.00 [0.00-0.00]  0.06 [0.03-0.09]/ 0.01 [0.00-0.02] | 0.88  0.10 | 1.00  0.71 |
|  | GG+GT  TT | 168/ 90  166/ 110 | 0.10 [0.04-0.15]/ 0.01 [0.00-0.03]  0.11 [0.05-0.16]/ 0.01 [0.00-0.02] | 0.12  **0.04** | 0.94  0.23 | 0.05 [0.01-0.10]/ 0.01 [0.00-0.03]  0.06 [0.02-0.10]/ 0.01 [0.00-0.02] | 0.43  0.23 | 1.00  0.76 |
| *VEGFA* rs699947 | AA  CA+CC | 28/ 25  306/ 175 | 0.04 [0.00-0.13]/ 0.00 [0.00-0.00]  0.11 [0.06-0.15]/ 0.01 [0.00-0.02] | 0.96  **0.004** | 1.00  0.91 | 0.04 [0.00-0.13]/ 0.00 [0.00-0.00]  0.06 [0.03-0.09]/ 0.01 [0.00-0.02] | 0.91  0.10 | 1.00  0.98 |
|  | AA+CA  CC | 180/ 104  154/ 96 | 0.11 [0.05-0.17]/ 0.00 [0.00-0.00]  0.09 [0.04-0.14]/ 0.02 [0.00-0.04] | **0.013**  0.27 | 0.99  0.65 | 0.06 [0.02-0.10]/ 0.00 [0.00-0.00]  0.05 [0.01-0.10]/ 0.02 [0.00-0.04] | 0.14  0.59 | 1.00  0.85 |
| *VEGFA* rs2010963 | CC  CG+GG | 63/ 34  271/ 166 | 0.09 [0.01-0.16]/ 0.03 [0.00-0.08]  0.11 [0.06-0.15]/ 0.01 [0.00-0.02] | 0.79  **0.004** | 1.00  0.58 | 0.04 [0.00-0.10]/ 0.03 [0.00-0.08]  0.06 [0.03-0.09]/ 0.01 [0.00-0.02] | 0.99  0.06 | 1.00  0.76 |
|  | CC+CG  GG | 219/ 139  115/ 61 | 0.13 [0.07-0.18]/ 0.01 [0.00-0.03]  0.06 [0.00-0.11]/ 0.00 [0.00-0.00] | **0.003**  0.65 | 0.98  0.89 | 0.07 [0.03-0.11]/ 0.01 [0.00-0.03]  0.04 [0.00-0.08]/ 0.00 [0.00-0.00] | 0.12  0.67 | 1.00  0.82 |

Value in bold indicate *p* < 0.05. **p* value, Adjusted by competitive years.

*COL3A1*, collagen III α1; *COL5A1*, collagen V α1; *COL12A1*, collagen XII α1; *MMP3*, matrix metalloproteinase 3; *DCN*, decorin; *ACAN*, aggrecan; *VEGFA*, vascular endothelial growth factor A
